# TSSK4 upregulation in alveolar epithelial type-II cells facilitates pulmonary fibrosis through HSP90-AKT signaling restriction and AT-II apoptosis

**DOI:** 10.1038/s41419-021-04232-3

**Published:** 2021-10-13

**Authors:** Huifang Chen, Andong He, Haoyang Li, Honglv Chen, Huancheng Xie, Liping Luo, Yuyi Huang, Jiaqian Chen, Jieying Guan, Qiaoling He, Jianjuan Ma, Changxing Ou, Ailin Tao, Jie Yan

**Affiliations:** 1 The Second Affiliated Hospital, The State Key Laboratory of Respiratory Disease, Guangdong Provincial Key Laboratory of Allery & Clinical Immunology, Guangzhou Medical University, Guangzhou, Guangdong 510260 China; 2 The First Affiliated Hospital, The State Key Laboratory of Respiratory Disease, Guangzhou Medical University, Guangzhou, Guangdong 510120 China; 3 Department of Pediatric Hematology, Affiliated Hospital of Guizhou Medical University, Guiyang, Guangxi 550000 China

**Keywords:** Apoptosis, Stress signalling

## Abstract

Alveolar epithelial injury is one of the important pathological changes in idiopathic pulmonary interstitial fibrosis (IPF), but the regulatory mechanism remains unclear. Here, we reported that alveolar epithelial type-II cells (AT II) play important roles in pathological process of pulmonary fibrosis. Through iTRAQ (isobaric tagging for relative and absolute quantification) quantitative proteomics, TSSK4 was identified to be upregulated in bleomycin-induced fibrotic mice model, which was further confirmed in clinical IPF patients’ tissue specimens. TSSK4 is a germ-related protein, but its expression in other tissues and the association with other diseases are not reported. Immunofluorescence staining showed that TSSK4 selectively expressed in AT-II cells, which are essential for inflammation-induced AT-II loss during fibrosis. Luciferase assay and other molecular biological experiments proved that TSSK4 expression is regulated by TNF-α-mediated NF-κB signaling. The TSSK4 kinase activity is found to be closely related to the function of HSP90-AKT pathway that TSSK4 can phosphorylate its substrate HSP90β on serine 255, to inhibit the ATPase activity of HSP90β and reduce its molecular chaperone function on AKT. Under this condition, kinase activity of AKT is diminished to interfere its survival function, subsequently facilitating AT-II cellular apoptosis through the mitochondrial death machinery. Our findings highlight the importance of TSSK4 in regulating pulmonary fibrosis by facilitating AT-II loss through HSP90-AKT signaling, all of which suggest TSSK4 and the regulating mechanism as attractive targets for the clinical intervention of pulmonary injury and fibrosis.

## Introduction

Idiopathic pulmonary fibrosis (IPF) is a kind of severe respiratory disease with high lethality but limited treatment, and the etiology still remains unclear [1, 2]. The pathological features of IPF include excessive extracellular-matrix (ECM) deposition, honeycomb changes, interstitial scarring, and fibroblast foci, all of which ultimately lead to pulmonary-structure destruction and irreversible loss of lung function [3, 4]. The prevalence of IPF appears to be increasing [5–7]. It is imperative to further investigate the molecular regulatory mechanism of pulmonary epithelial injury and pulmonary fibrosis, as well as early intervention in therapeutic approaches.

Alveolar epithelial cells play an important role in maintaining the normal lung function [8]. The pulmonary alveolar epithelium is mainly composed of two types of epithelial cells: alveolar type-I (AT I) and type-II (AT-II) cells. AT-II cells are considered to be progenitor (stem) cells of alveolar epithelium, which are responsible for epithelium regeneration after injury [9, 10]. It is reported that the pathological process of IPF involves repeated epithelial injury and abnormal damage repair, subsequently leading to the activation of intestinal fibroblasts, collagen deposition, and development of fibrotic lesions [11, 12]. It is reported that the function of AT II is impaired during pulmonary fibrosis [13, 14]. Nevertheless, it remains unclear how the AT-II dysregulation is triggered and what is the mechanism for AT II involving in IPF.

Testis-specific serine kinase 4 (TSSK4) is a member of the testis-specific serine kinases’ family. It is reported to mainly express in the acrosome part of sperm and participate in the formation of normal structure of sperm tail [15, 16]. Studies have shown that TSSK4 can induce apoptosis in some cell lines in vitro, such as HeLa, COS-7, and H1299 cell lines, and its pro-apoptotic function was verified to be related to its protein-kinase activity [17]. Whether TSSK4 is expressed in other specific organizations to participate in other functions needs further verification.

AKT, also known as protein-kinase B (PKB), is involved in cell survival by inhibiting the apoptotic process [18]. AKT could phosphorylate BAD (BCL2 antagonist of cell death), a pro-apoptotic protein of the BCL-2 family, to block the translocation of BAD to mitochondria and consequently interfere the mitochondrial apoptotic machinery [19]. Heat-shock protein 90 (HSP90), including two isoforms α and β, is a widely existing and highly conserved molecular chaperone to assist its “client” proteins for stabilization and proper performance of protein functions [20, 21]. HSP90 protein is involved in many cellular behaviors, including cell cycle regulation, hormone-signal transduction, and cell homeostasis [22, 23]. HSP90 is reported to stabilize some signaling molecules, including PI3K and AKT proteins. Inhibition of HSP90 could restrict PI3K/AKT pathway, leading to downregulation of survival function and resulting in apoptosis of target cells [24].

In this article, based on high-throughput proteomic analysis, we report that TSSK4 is selectively expressed in AT II and promotes AT-II loss through HSP–AKT signaling axis during pulmonary fibrosis. It is one of the molecular mechanisms for the process of pulmonary fibrosis, in which interfering the expressing of TSSK4 or regulating the activity of AKT could be the potential therapeutic strategies to improve pathological process of IPF.

## Results

### TSSK4 is upregulated in type-II alveolar epithelial cells in bleomycin-induced pulmonary fibrosis

**Fig. 1:**
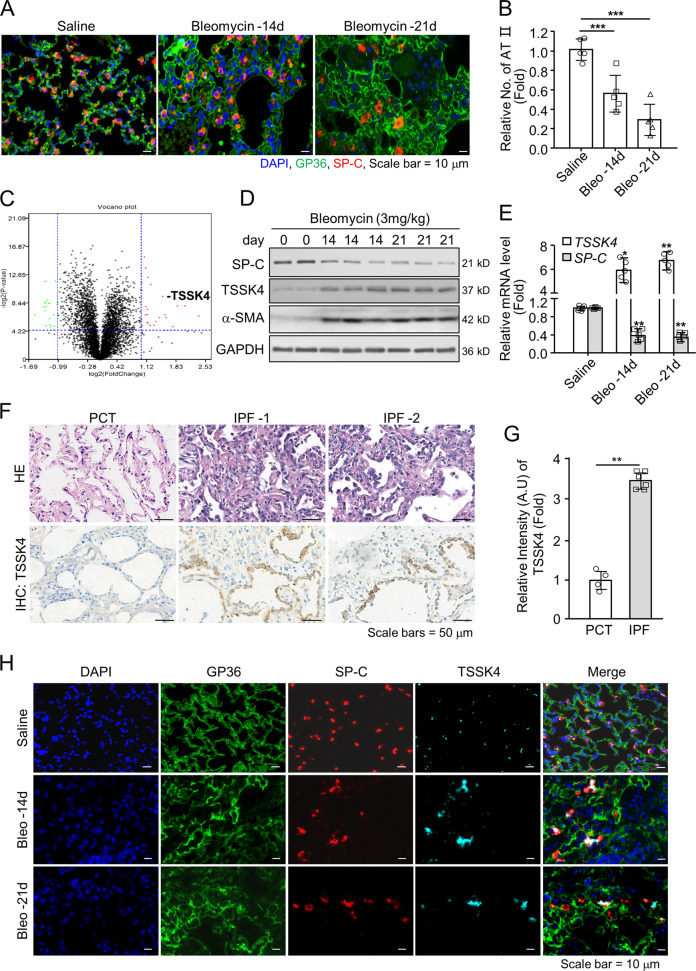
TSSK4 is selectively upregulated in AT-II cells of pulmonary fibrosis. C57BL/6 mice were intratracheally treated with bleomycin (3 mg/kg body weight) or the same amount of saline for various durations as indicated. **A**, **B** Representative lungs were immunofluorescence-stained with anti-GP36 antibody for AT-I cells, anti-SP-C for AT-II cells, and DAPI for nuclei (**A**); five individual mice of each group with three random fields of (**A**) were performed to analyzed the relative number of AT-II cells in the alveolar area through ImageJ program. The scatterplot represents the average value of each mouse (**B**). **C** Protein levels were measured by quantitative proteomics analysis. TSSK4 was indicated in the volcano plot (*n* = 3/group). **D** Immunoblot analysis of TSSK4, SP-C, and α-SMA level in the lungs of fibrosis models at different time points as indicated, with GAPDH as internal control. **E** Quantitative mRNA expression of *TSSK4* and *SP-C* genes in the lungs of fibrosis models as (**A**) was detected through qPCR (*n* = 5/group). **F**, **G** Lung tissues from clinical IPF patients (*n* = 6) were analyzed by H&E and IHC staining with anti-TSSK4 antibody. Paracancerous lung tissue (PCT, *n* = 4) was used as negative control (**F**); at least five random fields of each sample from (**F**) were performed to analyze the relative signal intensity of TSSK4 through ImageJ program. The scatterplot represents the average value of each sample (**G**). **H** Localization of TSSK4 was detected through immunofluorescence staining in representative lungs of fibrosis models as (**A**). AT I, AT II, and nuclei were separately stained with anti-GP36, anti-SP-C antibodies, and DAPI. Data in (**B**, **E**, **G**) are presented as mean ± s.d. *p < 0.05, **p < 0.01, ***p < 0.001, as analyzed by two-tailed unpaired Student’s *t* test. All data represent 2–3 individual experiments with similar results.

To investigate the pathological progress of pulmonary fibrosis, we utilized a relatively mild-grade mice model that C57BL/6 mice were intratracheally challenged with 3 mg/kg body weight of bleomycin. General observation found that without obvious mortality, the body weight of the model mice decreased dramatically in the first 14 days after bleomycin induction, and gradually increased with the passage of time (Fig. S1A). Nevertheless, HE staining and Masson’s trichrome staining showed significant pulmonary tissue damage, collagen deposition and fibrotic response on days 14 and 21 (Fig. S1B, C), with significantly increased RNA level of fibrosis-related genes (Fig. S1D). Moreover, immunofluorescence staining showed the number of type-II alveolar epithelial cells (AT II, SP-C-positive staining) in the area of fibrotic lung tissue (type-I alveolar epithelial area with GP36-positive staining) observably decreased (Fig. 1A, B). In order to explore the key proteins that are dysregulated in the fibrotic lung tissue, lung tissues from bleomycin-induced mice models were analyzed by iTRAQ (isobaric tagging for relative and absolute quantification) quantitative proteomics (data are available via ProteomeXchange with identifier PXD027900). The results showed that in AT-II-associated proteins (LAMP3, ABCA3, and SFTPC), the expression of ABCA3 and SFTPC was significantly downregulated, which can also suggest the loss of AT II in pulmonary fibrosis (Fig. S1F). According to the analysis of iTRAQ, TSSK4 was detected to be upregulated (Fig. S1E, C). Based on the analysis of human protein Atlas database, it is worth noting that the RNA and protein of TSSK4 can also be detected in human lung tissues (Fig. S1G). The expression of TSSK4 in other tissues was also confirmed accordingly (Fig. S1H, I). We further confirmed the finding by immunoblotting and qPCR that although the protein and RNA level of TSSK4 were low in the resting condition, it significantly increased on days 14 and 21 of bleomycin induction, suggesting TSSK4 to be a kind of inducible protein in fibrotic lung tissues (Fig. 1D, E). It was further verified in the clinical human samples by immunohistochemistry analysis that compared with the paracancerous pulmonary tissues as control, the protein level of TSSK4 was significantly upregulated in clinical fibrotic lung tissues (in total, six fibrotic patient samples were tested with similar results, two of which are presented in Fig. 1F, G. Demographic information and the results of clinical examination are listed in Table 1). We further investigated the specificity of TSSK4 in lung tissue by immunofluorescence staining and the results showed that upregulated TSSK4 was mainly localized in AT-II cells (Fig. 1H), which strongly suggested that the upregulation of TSSK4 selectively occurred in AT-II cells.

**Table 1:** Demographic information and clinical examination on six IPF patients from whom biopsy specimens were obtained.

No.	Age	Gender	FVC (%)	DLCO (%)	WBC (*10^9^/l)	NEU (%)	LYM (%)	MON (%)	EOS (%)	BAS (%)
**1**	63	Male	142.5	82.3	5.9	64	20	10.6 ↑	5	0.5
**2**	59	Male	72 ↓	45 ↓	9.5	59	28.5	9.8 ↑	2.4	0.3
**3**	46	Male	79.58 ↓	96.9	10.25 ↑	67.8	25.6	6.4	0	0.1
**4**	38	Male	56 ↓	61 ↓	7.07	66.6	24.2	6.6	2	0.6
**5**	52	Female	94	74	5.3	65.9	20.8	11.0 ↑	1.9	0.4
**6**	46	Female	58.5 ↓	51.6 ↓	4.61	65.6	21.6	10.4 ↑	0.5	0

*FVC* forced vital capacity, *DLCO* diffusing capacity of the lung for carbon monoxide, *WBC* white blood cells, *NEU* neutrophil, *LYM* lymphocyte, *MON* monocyte, *EOS* eosinophil, *BAS* basophil, ↑ increase, ↓ decrease.

### TSSK4 upregulation is essential for bleomycin-induced pulmonary fibrosis

**Fig. 2:**
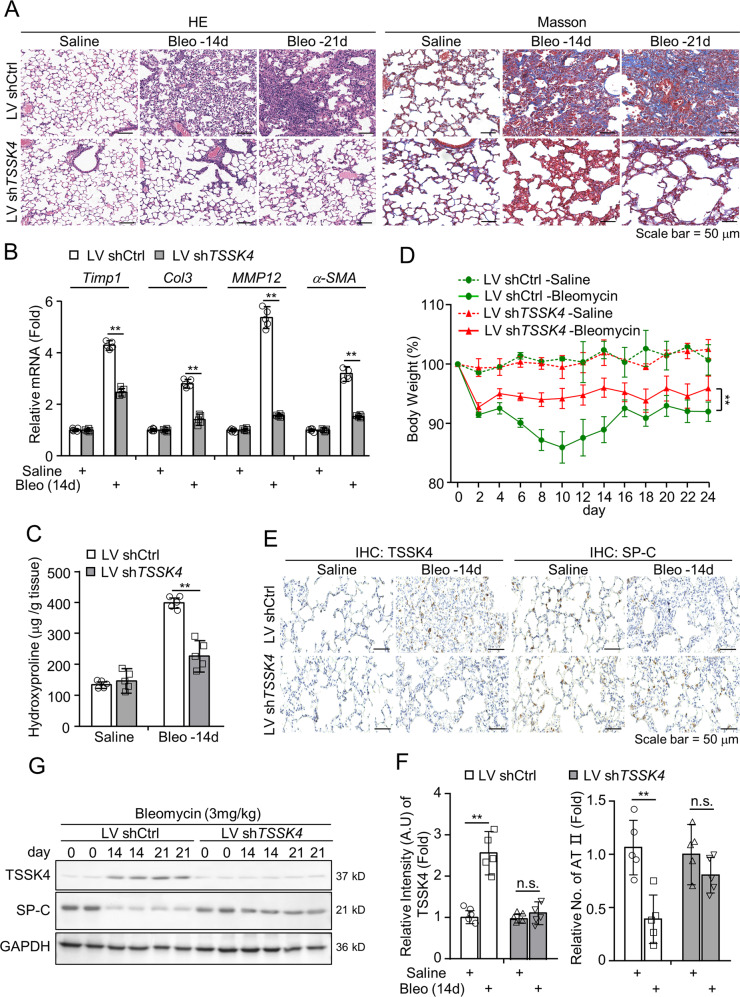
TSSK4 knockdown ameliorates bleomycin-induced lung fibrosis. C57BL/6 mice were intratracheally infected with lentivirus-mediated sh*TSSK4* or control vectors (1.05 × 10^10^ infectious units (IFUs) in a volume of 30 μl per animal), and then intratracheally treated with bleomycin (3 mg/kg body weight) or the same amount of saline for a period as indicated. **A** H&E staining and Masson’s trichrome staining of the representative lungs from fibrosis model mice as indicated. **B** Quantitative mRNA expression of the fibrotic genes, including *Timp1*, *Col3*, *MMP12*, and *α-SMA* in the lungs of fibrosis models as (**A**) was detected through qPCR (*n* = 5/group). **C** Hydroxyproline assay of the lungs from different groups as indicated was performed (*n* = 5/group). **D** Relative body-weight loss of model mice was detected for a period of 24 days (*n* = 10/group). **E**, **F** IHC staining with anti-TSSK4 and anti-SP-C antibodies in the representative lungs from fibrotic model mice (**E**); five individual mice of each group with three random fields of (**E**) performed to analyze the relative signal intensity of TSSK4 and number of AT-II cells in the alveolar area through ImageJ program. The scatterplot represents the average value of each mouse (**F**). **G** Immunoblot analysis of TSSK4 and SP-C level in the lungs of different fibrotic groups at different time points as indicated, with GAPDH as internal control. Data in (**B**, **C**, **D**, **F**) are presented as mean ± s.d. In (**B**, **C**, **F**), **p < 0.01, n.s. *p* > 0.05, as analyzed by two-tailed unpaired Student’s *t* test. In (**D**), **p < 0.01, was analyzed by one-way ANOVA test. All data represent 2–3 individual experiments with similar results.

To investigate the contributions of TSSK4 upregulation in lung fibrosis, a vector of small-hairpin RNA targeting *TSSK4* was imported into C57BL/6 mice by lentivirus-mediated intrapulmonary infection, to suppress TSSK4 expression in vivo (Fig. 2G, first panel). Pathological examination showed that compared with the control group, lung damage and collagen aggregation were significantly reduced in *TSSK4*-interference group (Figs. 2A and S2). It was also confirmed that *TSSK4* knockdown distinctly reduced fibrotic responses, such as less induction of pulmonary fibrotic genes (Fig. 2B), less hydroxyproline in tissues (Fig. 2C), and statistically significant improvement of the symptoms of weight loss in fibrotic mice models (Fig. 2D). Immunohistochemistry and immunoblotting analysis showed that TSSK4 depletion could significantly reduce the loss of AT II in pulmonary tissue (Fig. 2E, G). Taken together, in fibrotic lung tissue, TSSK4 is selectively upregulated in AT-II cells, thereby promoting the loss of AT II and pathological progress of pulmonary fibrosis.

### TSSK4 upregulation is regulated by NF-κB signaling pathway

**Fig. 3:**
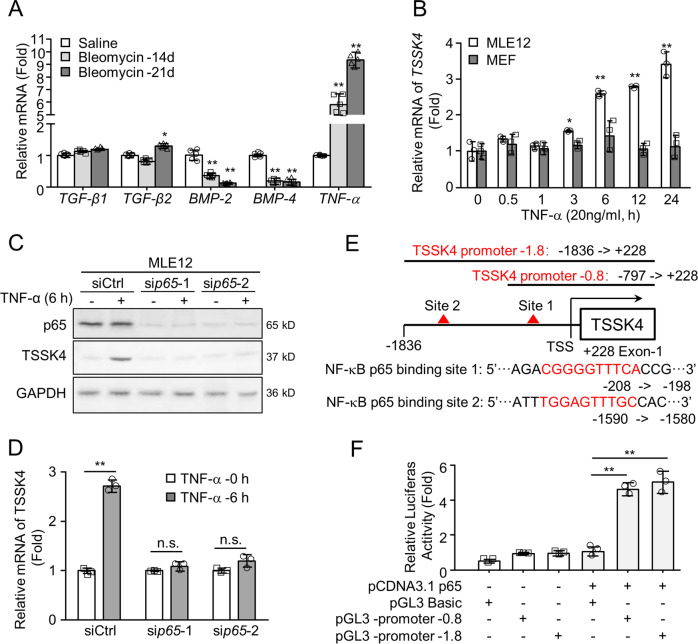
TSSK4 expression is regulated by NF-κB pathway. **A** Quantitative mRNA expression of the indicated genes in lung tissues of fibrosis models as (Fig. 1A) was detected through qPCR (*n* = 5/group). **B** MLE-12 cells and MEFs (mouse embryonic fibroblasts) were stimulated with TNF-α (20 ng/ml) for various durations, as indicated. Relative mRNA expression of *TSSK4* was detected through qPCR analysis. **C**, **D** MLE-12 cells were transfected with scramble siRNA (siCtrl) or two different target sites of si*p65*, followed by treatment without or with TNF-α (20 ng/ml) for 6 h. Protein levels of TSSK4 and P65 were analyzed by immunoblotting, with GAPDH as internal control (**C**); relative mRNA expression of TSSK4 was detected through qPCR analysis (**D**). **E** Nucleotide schematic diagram of predicted potential p65-binding sites in the TSSK4-promoter region. TSS, transcript start site was set as +1 nucleotide. Sequences of potential binding sites were listed with nucleotide position relative to TSS. Two promoter fragments (−1.8 and −0.8) included different lengths of 5′-flanking region and the first exon in the 3′-region as indicated. **F** Two promoter fragments were constructed into the pGL3-basic vector, combined with p65-expression pCDNA3.1 vector, and cotransfected into 293T cells separately as indicated. Normalized firefly luciferase activity in the cellular lysates from each construct was examined. Data in (**A**, **B**, **D**, **F**) are presented as mean ± s.d. **p < 0.01, n.s. *p* > 0.05, as analyzed by two-tailed unpaired Student’s *t* test.

To further investigate the upstream-regulatory mechanism of TSSK4 expression, we detected cytokines in pulmonary fibrotic mice tissues. The qPCR results showed that the mRNA level of TNF-α was significantly increased, but not TGF-β1 or TGF-β2 (Fig. 3A). To explore whether TNF-α was the key cytokine to promote TSSK4-selective expression in AT II, MLE12 (commonly used murine cell line of AT II) was performed to be stimulated with TNF-α (20 ng/ml). According to the stimulating time course, *TSSK4* transcription gradually increased in MLE12, but not in MEFs (mouse embryonic fibroblasts) (Fig. 3B). Silencing of *p65* in MLE12 inhibited TNF-α-induced TSSK4 expression both in protein and mRNA levels (Fig. 3C, D). It is consistent with the results of inhibitor treatment that NF-κB inhibitor (Bay 11–7082) but not P38 inhibitor (SB202190) could effectively block TSSK4 upregulation (Fig. S3A). All of these results suggest that TNF-α activated NF-κB to be the possible regulator of TSSK4 expression.

In order to further confirm the regulation of transcription factor p65 on *TSSK4* gene expression, we analyzed the gene sequence of human *TSSK4* (Gene ID: 283629) to identify the 5′-flanking region. Computational prediction indicated two potential p65-binding sites in the promoter region (about 1.8 kb up to transcript start site (TSS)) (Figs. 3E and S3B). As presented in the nucleotide schematic diagram, two truncated promoter fragments were constructed for dual-luciferase assay. The results showed that compared with full length of promoter region (−1.8 kb), the 5′- flanking region containing −0.8 kb is sufficient for maximal activity, suggesting it to be the major p65-bind region for *TSSK4* gene transcription (Fig. 3F). The exact p65-binding nucleotide sequence requires further site mutation and report gene-activity analysis.

Taken together, our results indicate that TSSK4 expression is regulated by TNF-α-promoted NF-κB signaling pathway, and the p65-binding sites locate in the 5′-flanking region −0.8 kb of *TSSK4*-promoter region.

### TSSK4 promotes AT-II loss through TNF-α-induced cellular apoptosis

**Fig. 4:**
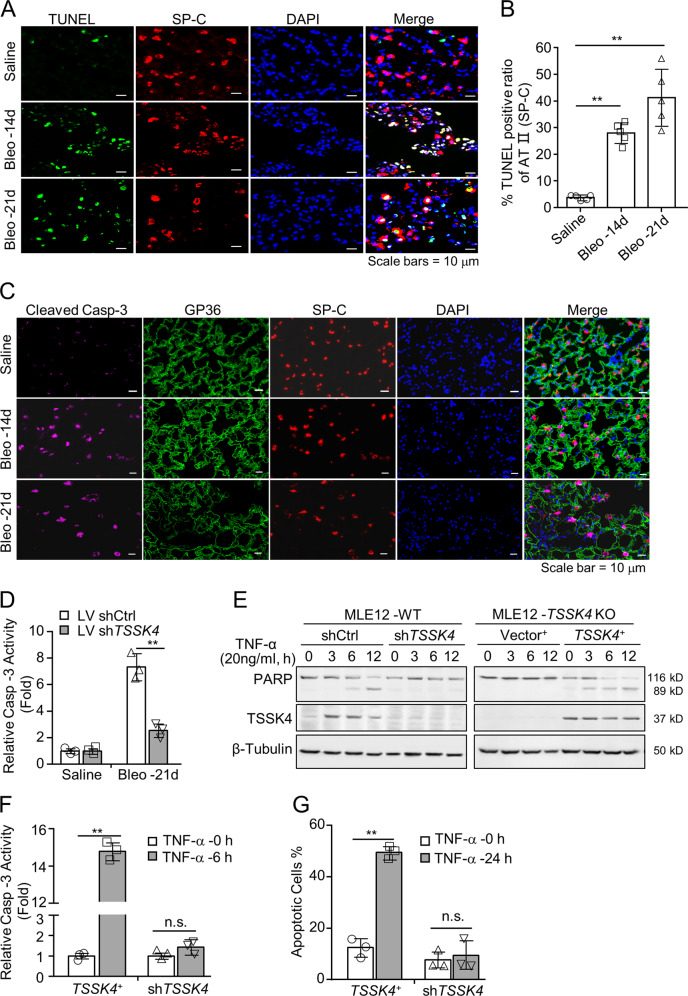
TSSK4 promotes AT-II loss through cellular apoptosis. **A**–**C** Representative lung sections from fibrotic models as in Fig. 1A were subjected to FITC-TUNEL staining and AT II co-immunofluorescence stained with anti-SP-C antibody. Nuclei were detected with DAPI (**A**); five individual mice of each group with three random fields of (**A**) performed to analyze the TUNEL-positive ratio in total AT-II cells by ImageJ program. The scatterplot represents the average value of each mouse (**B**); representative lung sections were co-immunofluorescence stained with anticleaved caspase-3 antibody, anti-GP36 for AT I, anti-SP-C antibody for AT II, and DAPI for nuclei (**C**). **D** Representative lungs from lentivirus-mediated interfering TSSK4 expression and bleomycin-induced fibrosis models as in Fig. 2 were subjected to caspase-3 activity analysis. **E**–**G** MLE-12 wild-type cells were transiently transfected with sh*TSSK4* or scramble shRNA (shCtrl) as control. In the *TSSK4* knockout cells that MLE-12 stably transfected with sh*TSSK4*, expression vector (*TSSK4*^+^) was transfected to mediate TSSK4-persistent expression. Empty vector (*Vector*^+^) was used as control. Different cells were stimulated without or with TNF-α (20 ng/ml) for various durations as indicated. Protein levels of TSSK4 and PARP with cleavage part were analyzed by immunoblotting, with α-tubulin as internal control (**E**); caspase-3 activity in cellular lysis of sh*TSSK4* and *TSSK4*^+^ was analyzed (**F**); apoptotic cells were detected by Annexin V/propidium iodide (PI) staining and analyzed by flow cytometry (**G**). Data in (**B**, **D**, **F**, **G**) are presented as mean ± s.d. *p < 0.01, **p < 0.01, n.s. *p* > 0.05, as analyzed by two-tailed unpaired Student’s *t* test. All data represent 2–3 individual experiments with similar results.

Based on the results of quantitative proteomics (Fig. 1C), KEGG signaling pathway enrichment analysis revealed apoptosis-related signaling pathway to be activated (Fig. S4A). We hypothesized that the loss of AT-II cells in fibrosis might be through apoptosis. Immunofluorescence staining showed that according to the extension of bleomycin induction, the TUNEL-positive signals in lung sections gradually increased, which were mainly concentrated in AT-II cells (Figs. 4A, B and S4B). It was consistent with another immunofluorescence staining result that cleaved caspase-3 also concentrated on AT-II but not on AT-I cells (Fig. 4C). All of these results suggested apoptosis of AT-II cells during the pulmonary fibrotic process.

Caspase-3 activity assay showed that lentivirus-mediated sh*TSSK4* induction could significantly inhibit caspase-3 activity in fibrotic lung tissues (Fig. 4D). In vitro results showed that TNF-α could induce PARP cleavage in wild-type MLE12 cells (Fig. S4C), but less cleaved PARP was detected when TSSK4 expression was suppressed by sh*TSSK4* interfering (Fig. S4D and left part of Fig. 4E). On the contrary, TNF-α induced a more significant PARP cleavage in TSSK4-persistent expressing MLE12 cells (*TSSK4*^+^) than that in wild-type or *TSSK4*-deficient cells (Fig. 4E). Consistently, TNF-α could promote caspase activity and cellular apoptosis in *TSSK4*^+^ cells, which were both suppressed in *TSSK4*-deficient cells (Fig. 4F, G).

Taken together, TSSK4 upregulation in AT-II cells plays an important role in TNF-α-induced cellular apoptosis in vitro and AT-II loss in pulmonary fibrosis.

### TSSK4 mediates AT-II apoptosis through the mitochondrial death machinery

**Fig. 5:**
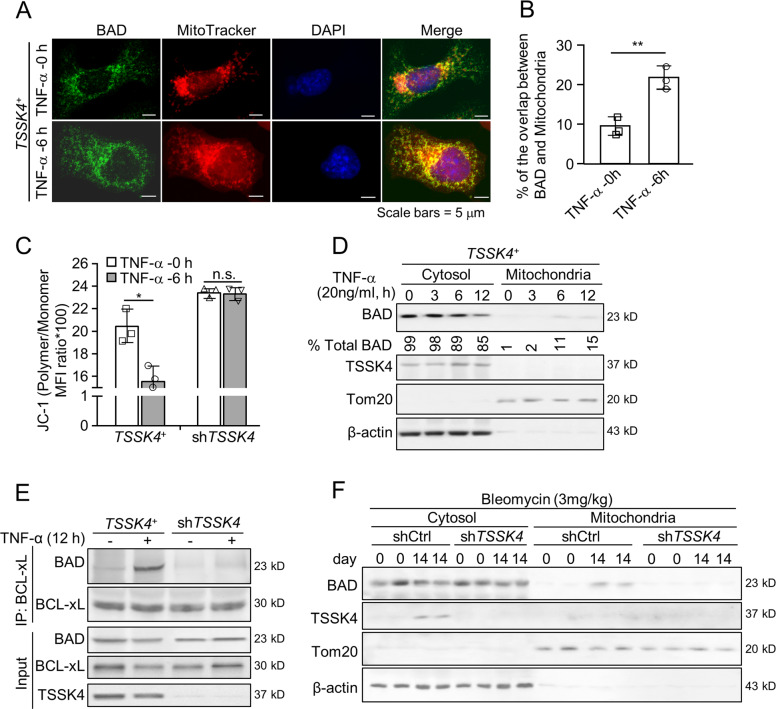
TSSK4 mediates AT-II apoptosis through the mitochondrial death machinery. **A**–**E** TSSK4-persistent expressing cells (*TSSK4*^*+*^, as mentioned in Fig. 4E) were stimulated without or with TNF-α (20 ng/ml) for various durations as indicated. Colocalization of BAD with mitochondria was analyzed by immunofluorescence staining with anti-BAD antibody, MitoTracker for mitochondria, and DAPI for nuclei, followed with confocal microscope (LSM880) observation (**A**); the ratio of BAD signaling that overlapped with mitochondria was quantitatively analyzed through ImageJ program in three independent experiments with five random-view fields of each treatment. The scatterplot represents the average value of each mouse (**B**); cells were stained with JC-1 dye, followed with flow-cytometric analysis to detect the relative mitochondrial membrane potential (**C**); cellular lysis was separated into cytosol and mitochondrial fractions. Sublocalization of BAD and TSSK4 level was detected by immunoblotting. β-Actin and Tom20 were used as cytosol and mitochondria markers, respectively. The amount of BAD in cytosol and mitochondrial fractions were quantitated by ImageJ program (**D**); protein interaction between BCL-xL and BAD was analyzed by immunoprecipitation with anti-BCL-xL antibody, followed by immunoblotting with relative antibodies separately. Input proteins were detected by immunoblotting in total cellular lysis (**E**). **F** Lung tissues from lentivirus-mediated interfering TSSK4 expression and bleomycin-induced fibrosis models as (Fig. 2) were separated into cytosol and mitochondrial fractions. Sub-localization of BAD and TSSK4 level were detected by immunoblotting. β-Actin and Tom20 were used as cytosol and mitochondria markers, respectively. Data in (**B**, **C**) are presented as mean ± s.d. *p < 0.01, **p < 0.01, n.s. *p* > 0.05, as analyzed by two-tailed unpaired Student’s *t* test. All data represent 2–3 individual experiments with similar results.

It is reported that TNF-α cytotoxicity in diseases depends on the BH3-only protein BAD-mediated mitochondrial death machinery [19, 25]. In TSSK4-persistent expressing MLE12 cells (*TSSK4*^+^), immunofluorescence staining showed that TNF-α promoted the translocation of BAD protein to mitochondria (Figs. 5A, B and S5). Furthermore, mitochondrial membrane potential decreased significantly under TNF-α induction, which was totally suppressed in TSSK4-deficient MLE12 cells (Fig. 5C). Mitochondria/cytosol fractionation showed that in *TSSK4*^+^ cells, TNF-α promoted the percentage of BAD in the mitochondrial fractionation (Fig. 5D). Immunoprecipitation results also confirmed that TNF-α induced the interaction between BAD and mitochondrial outer-membrane protein BCL-xL in *TSSK4*^+^ cells but not in *TSSK4*-deficient cells (Fig. 5E). The same results further presented that BAD translocated to mitochondria in lung tissues of bleomycin-induced fibrosis, which was dramatically suppressed in lentivirus-mediated *TSSK4*-deficient model mice (Fig. 5F).

Taken together, AT-II apoptosis depends on the mitochondrial death machinery. Interfering TSSK4 expression could block the translocation of BAD to mitochondria, thereby inhibiting its pro-apoptotic function.

### TSSK4 restrains AKT activation and consequently promotes AT-II apoptosis in vitro and in vivo

**Fig. 6:**
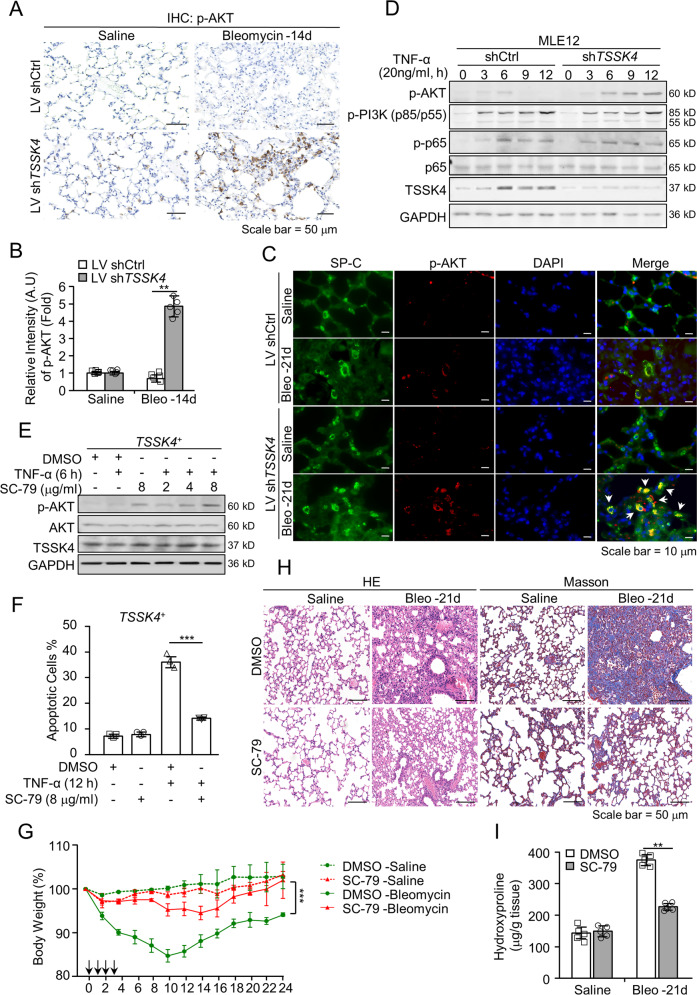
TSSK4 restrains AKT activation and consequently promotes AT-II apoptosis in vitro and in vivo. **A**–**C** Representative lungs from lentivirus-mediated interfering TSSK4 expression and bleomycin-induced fibrosis models as (Fig. 2) were analyzed by IHC staining with anti-p-AKT antibody (**A**); at least five random fields of (**A**) were performed to analyze the relative signal intensity of p-AKT through ImageJ program (**B**); sublocalization of p-AKT was detected through immunofluorescence staining with anti-p-AKT antibody, anti-SP-C antibody for AT II, and DAPI for nuclei separately (**C**). **D** MLE-12 wild-type cells were transiently transfected with sh*TSSK4* or scramble shRNA (shCtrl) as control, and stimulated without or with TNF-α (20 ng/ml) for various durations as indicated. Phosphorylation of AKT and PI3K, as well as their original protein levels and TSSK4 were analyzed by immunoblotting with corresponding antibodies. GAPDH was detected as internal control. **E**, **F** *TSSK4*^*+*^ cells (as mentioned in Fig. 4E) were pretreated with DMSO or different doses of SC-79 (for AKT activation) as indicated for 1 h, followed by stimulation without or with TNF-α (20 ng/ml) for 6 or 12 h, as indicated. AKT phosphorylation with its original protein level and TSSK4 were detected by immunoblotting (**E**); apoptotic cells were detected by Annexin V/propidium iodide (PI) staining and analyzed by flow cytometry (**F**). **G**–**I** C57BL/6 mice were induced lung fibrosis with bleomycin as (Fig. 1A). Model mice were daily intraperitoneally injected with SC-79 (40 mg/kg body weight in 10% DMSO) or the same amount of DMSO for the first 3 days. Relative body-weight loss of model mice was detected for a period of 24 days (*n* = 8/group) (**G**); representative lungs on day 21 were performed by H&E staining and Masson’s trichrome staining, respectively (**H**); hydroxyproline assay of the indicated lung lysis was analyzed (*n* = 5/group) (**I**). Data in (**B**, **F**, **G**, **I**) are presented as mean ± s.d. In (**B**, **F**, **I**), **p < 0.01, ***p < 0.001, as analyzed by two-tailed unpaired Student’s *t* test. In (**G**), ***p < 0.001 by one-way ANOVA test.

It was reported that PI3K–AKT pathway plays an essential role in cellular survival [26]. IHC staining showed that compared with the control group, the level of phosphorylated AKT in bleomycin-induced fibrotic lung tissues was significantly upregulated when TSSK4 was depleted (Figs. 6A, B and S6A). TSSK4 depletion could significantly facilitate AKT phosphorylation under fibrotic condition (Fig. S6B, second panel). Meanwhile, immunofluorescence staining showed that AKT phosphorylation mainly occurred in AT-II cells (Fig. 6C). With the activation of AKT, serine 136 of BAD (the specific phosphorylation site by AKT to negatively regulate the pro-apoptotic function of BAD [27]) was also increased (Fig. S6B, first panel), but apoptotic signaling (PARP cleavage) was decreased (Fig. S6B, fifth panel). All of these data suggest AKT signaling pathway to be closely related to AT-II loss in pulmonary fibrosis.

As presented in (Fig. 6D), TNF-α stimulation induced upregulation of TSSK4 in MLE12 cells, but AKT phosphorylation was very weak. On the other hand, when blocking TSSK4 with shRNA, the AKT activation but not PI3K or p65 increased. It suggests that TSSK4 expression could suppress AKT activation in cells. As our results showed, TNF-α-induced apoptosis of *TSSK4*^*+*^ was significantly reduced when AKT was activated by AKT agonist SC-79 (Fig. 6E, F). On the other hand, in *TSSK4*-deficient cells, AKT inhibitor GSK690693 promoted TNF-α-induced apoptosis (Fig. S6C, D). Together, our data demonstrate that TSSK4 upregulation could inhibit AKT activation, thereby impairing its prosurvival function.

Because of weak AKT activation in bleomycin-induced pulmonary fibrosis (Fig. 6A, B), we further used AKT agonist SC-79 to treat mice. The results showed that intraperitoneal administration of SC-79 in the first 3 days of bleomycin induction could improve the fibrotic symptoms, including tissue pathological changes, collagen deposition, and body-weight loss (Figs. 6G–I and S6F). In brief, the AKT agonist contributes to enhance the prosurvival function of AKT, thereby ameliorating bleomycin-induced fibrosis.

### TSSK4 phosphorylates HSP90β on serine 255

**Fig. 7:**
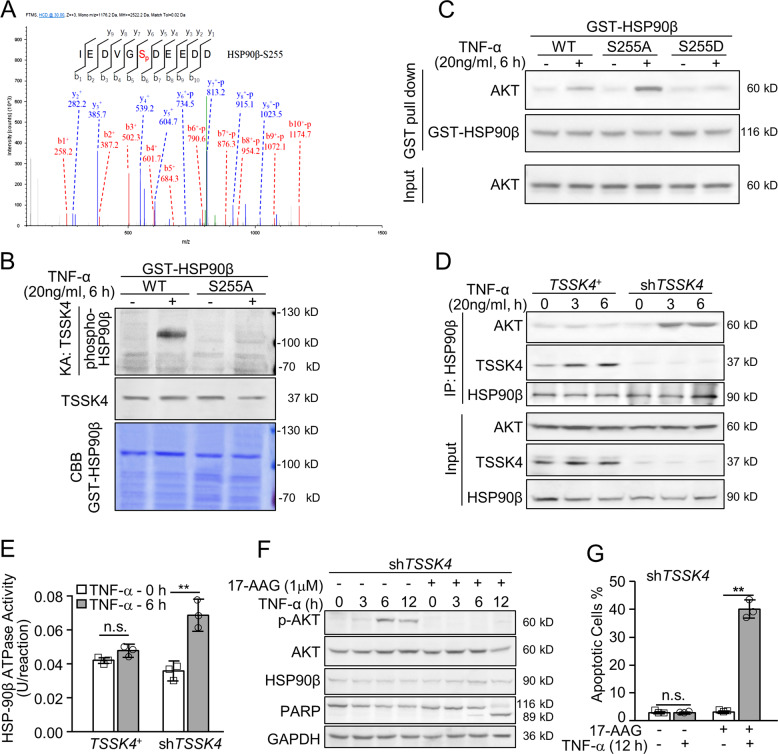
TSSK4 phosphorylates HSP90β to suppress HSP90–AKT signaling axis for AT-II apoptosis. **A** Lung-tissue lysis from fibrosis models as (Fig. 1A) was measured by quantitative phosphorylated proteomics analysis (*n* = 3/group). Mass spectrogram showed the recovered phosphorylated peptide fragment of HSP90β corresponding to S255. **B** *TSSK4*^*+*^ cells (as mentioned in Fig. 4E) were stimulated without or with TNF-α (20 ng/ml) for 6 h. Phosphorylation of GST-HSP90β (WT) or GST-HSP90β (S255A) mutant proteins by active TSSK4 was analyzed by relative purified GST substrates incubating with TSSK4 immune-complex kinase in the presence of nonradioactive ATP and detected by immunoblotting with antiserine phosphorylation antibody. Input GST proteins were detected with Coomassie brilliant blue staining (CBB). **C** MLE12 cells were stimulated without or with TNF-α (20 ng/ml) for 6 h. Cellular lysis was incubated with purified GST-HSP90β wild-type or mutant (S255A, S255D) proteins as indicated. The precipitated complexes, as well as AKT level in the input cellular lysis, were detected by immunoblotting with anti-AKT and anti-HSP90β antibodies. **D**, **E** *TSSK4*^*+*^ cells and *TSSK4* knockdown cells (sh*TSSK4*, as mentioned in Fig. 4E) were stimulated without or with TNF-α (20 ng/ml) for 6 h. The association among HSP90β, TSSK4, and AKT was analyzed by immunoprecipitation with anti-HSP90β antibody, followed by immunoblotting with relative antibodies separately (**D**); relative HSP90β ATPase activity was analyzed in immunoprecipitated HSP90β from total lysis as indicated (**E**). **F**, **G** sh*TSSK4* cells were pretreated without or with 17-AGG (1 μM, for HSP90 inhibition) for 1 h, followed by stimulation without or with TNF-α (20 ng/ml) for various durations, as indicated. AKT phosphorylation with its original protein level, TSSK4, HSP90β, and PARP was detected by immunoblotting (**F**); apoptotic cells were detected by Annexin V/propidium iodide (PI) staining and analyzed by flow cytometry (**G**). Data in (**E**, **G**) are presented as mean ± s.d. **p < 0.01, n.s. *p* > 0.05, as analyzed by two-tailed unpaired Student’s *t* test.

Since TSSK4 is a kind of serine-/threonine-phosphorylation kinase, we hypothesized that the mechanism of TSSK4-suppressing AKT activation was based on kinase-substrate reaction. In order to authenticate possible TSSK4-phosphorylation substrates and map out the precise phosphorylation site(s), lung tissues on day 14 of bleomycin induction were performed for iTRAQ quantitative-phosphorylation mass spectrometry. Phosphorylated HSP90β was detected with high detection ratio, and the phosphorylated site was serine 255 as the mass spectrogram presented (Fig. 7A). To further confirm HSP90β as the substrate of TSSK4, we replaced the predicted serine 255 with nonphosphorylated alanine using a site-directed mutagenesis approach. TSSK4 immune-complex kinase assay showed that TNF-α stimulation could promote TSSK4 to phosphorylate the GST-HSP90β (WT), but not GST-HSP90β (S255A) mutant (Fig. 7B). Together, HSP90β is the substrate of TSSK4 with serine 255 as the specific phosphorylation site.

### TSSK4 phosphorylates HSP90β to suppress HSP90–AKT signaling axis for AT-II apoptosis

GST pull-down experiments showed that TNF-α induced mild interaction between AKT and wild-type GST-HSP90β (Fig. 7C, left panels). This kind of AKT-HSP90β interaction was substantially increased when serine 255 of HSP90β mutated with nonphosphorylate alanine (S255A) (Fig. 7C, middle panels). On the contrary, AKT-HSP90β interaction was dramatically reduced when serine 255 mutated with phosphorylated mimic aspartic acid (S255D) (Fig. 7C, right panels). The results indicate that TSSK4 phosphorylating HSP90β on serine 255 could interfere its interaction with AKT. It was further proved by the immunoprecipitation results that TNF-α induced a more significant AKT-HSP90β interaction in *TSSK4*-deficient cells than that in TSSK4-persistent expressing MLE12 cells (*TSSK4*^+^) (Fig. 7D).

Because the chaperone function of HSP90β is in ATP-dependent manner, we next detected the effect of TSSK4 on the ATPase activity of HSP90. Our results showed that TNF-α could promote ATPase activity of HSP90β in *TSSK4*-deficient MLE12 cells, but no effect in *TSSK4*^+^ cells (Fig. 7E). Immunoblotting results showed that TNF-α could promote AKT phosphorylation and activation, consequently less PARP cleavage in *TSSK4*-deficient cells, all of which were totally reversed when cells were pretreated with HSP90 inhibitor 17-AAG (Fig. 7F). It was consistent with cellular apoptotic analysis that 17-AGG dramatically enhanced TNF-α-induced apoptosis in *TSSK4*-deficient MLE12 cells (Fig. 7G).

Taken together, TSSK4 expression could suppress the ATPase activity of HSP90, interfered its interaction with AKT, and thereby affects the activation of AKT and its prosurvival function.

## Discussion

**Fig. 8:**
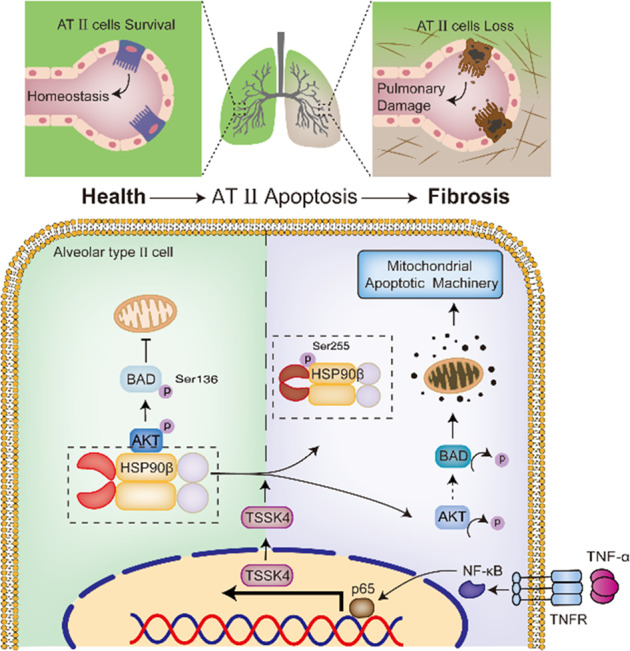
Graphical summary of cellular and molecular mechanism. A schematic presentation of the mechanism by which TSSK4 upregulation in AT II promotes AT-II loss via HSP90-AKT regulation and mitochondrial apoptotic machinery, resulting in pulmonary damage and fibrosis. See the text for details.

In this report, we demonstrate that TNF-α induces selective expression of TSSK4 in AT II cells, thereby facilitates the apoptotic pathway for AT II loss, local lung tissue damage and fibrosis as pathological characters. At the molecular level, TNF-α-mediated NF-κB signaling pathway initiates TSSK4 gene expression in AT II. TSSK4 can phosphorylate its substrate HSP90β on serine 255, to inhibit its ATPase activity and reduce its molecular chaperone function on AKT. Under this condition, kinase activity of AKT is diminished to interfere its survival function, that pro-apoptotic protein BAD, without its negative regulatory phosphorylation by AKT (serine 136), translocates to the outer membrane of mitochondria to promote apoptosis through the mitochondrial death machinery (Fig. 8).

The in situ staining and other cellular results further demonstrated that AT-II loss depends on mitochondrial apoptotic machinery (Figs. 4A, C and 5A). The qPCR results showed that the mRNA level of TNF-α was significantly increased (Fig. 3A), which suggests TNF-α to be the major trigger of AT-II apoptosis. According to literatures, besides TNF-α, TGF-β is also important for the development of IPF. TGF-β superfamily includes TGF-β1, TGF-β2, and bone morphogenic proteins (BMPs). It is reported that the crosstalk between TGF-β and BMPs plays important roles in fibrotic processes. In lung, BMP-4 and BMP-7 could play antergic function against TGF-β to improve fibrosis [28, 29]. Based on our results (Fig. 3A), various expression levels of TGF-β superfamily (TGF-β 1, TGF-β 2, BMP-2, and BMP-4) were detected, suggesting the complexity of TGF-β superfamily in fibrosis. Further research is required to investigate the feedback regulation of TGF-β superfamily and the crosstalk among different members for fibrotic progress.

TSSK4-mediated HSP90 suppression leads to AKT deactivation in AT II, which is one of the important mechanisms for AT-II loss in fibrosis (Fig. 8). AKT plays an important role in lots of cellular behaviors, such as cell survival, cell differentiation, cellular activation, etc., but the contribution of AKT pathway in fibrosis is complicated and remains to be further investigated. For lung fibrosis, AKT activation is reported in different cells with different pathological functions. For example, AKT activation in macrophages could promote several fibrotic factors for wound healing and organ fibrosis; AKT activation in fibroblasts could regulate collage expression [30]. From these perspectives, AKT inhibitors could be as potential therapeutic agents for IPF [30]. On the other hand, we found that AKT was suppressed selectively in AT-II cells because of TSSK4 expression that promoted AT-II loss under inflammatory conditions. It is convinced that AT-II loss happens at the early stage of IPF and further promotes pulmonary damage and subsequent fibrosis. Therefore, it is meaningful for AKT agonist application in the early stage of IPF to prevent AT-II loss. It is not contradicted with AKT-inhibitor application mentioned above, because both of them focus on different molecular mechanisms and targets in different pathological stages during the whole IPF progress. Our data showed that in vivo application of AKT agonist SC-79 has an obvious inhibitory effect on bleomycin-induced pulmonary fibrosis (Fig. 6G, I). According to our results, SC-79 treatment in the first 3 days has a significant effect on fibrosis inhibition (Fig. 6G, I), but the intervention effect is not obvious or even counterproductive if treated with SC-79 for more than 7 days (data not shown). We believe that the effect of SC-79 is focused on the apoptosis of AT II as the inhibitory target in the initial stage. The key point is to find more specific compounds/drugs to target on the core regulatory cells and processes. AT-II loss and pulmonary damage are the initiator of fibrosis but fibroblast activation involves in the subsequent pathological progress. AT-II protection and fibroblast suppression are two opposite and independent potential strategies for clinical treatments of IPF. Further in-depth research is required to investigate the efficacy of early application of AKT agonist (such as SC-79) combined with late-selective fibroblast suppression in pulmonary fibrosis therapeutic treatment.

Taken together, given the importance of TSSK4 upregulation in AT II for cellular apoptosis and thereby fibrosis, our finding is likely to have great significance for further understanding the pathogenesis of pulmonary fibrosis and subsequent clinical application of IPF.

## Materials and methods

### Reagents

Antibodies against TSSK4 (A7861) and caspase-3 (A0214) were purchased from ABclonal. Antibodies against HSP90β (GB12284) and PARP (GB111500) were from Servicebio. Antibody against Phospho-PI3-kinase p85/p55 (YP0224) was from ImmunoWay. Antibodies against SP-C (10774-1-AP), Tom20 (11802-1-ap), and p-AKT (66444-1-ig) were from Proteintech. Antibodies against p-BAD Ser136 (9295), BCL-xL (2762), and β-actin (c3700) were from CST. Antibodies against phosphoserine (ab9332), GP36 (ab11936), and p65 (ab16502) were from Abcam. Compounds SC-79 (T2274), Bay11-7082 (19524-67-7), SB202190 (152121-30-7), and 17-AAG (T6290) were from Target Mol. TNF-α (murine) was from R&D. JC-1 Assay Kit was from Molecular Probes of Life Technologies. Hydroxyproline (HYP) Assay Kit (20201102) was from Solarbio.

For *TSSK4* gene silence, small-hairpin RNA targeting *TSSK4* was constructed in pLV[shRNA]-EGFP: T2A: Puro vector. Three different target sequences are listed as:

sh*TSSK4*-1: CCACGTGAGATACAGGTAATG; sh*TSSK4*-2: CATCGTGCACCGGGATTTAAA; sh*TSSK4*-3: CTCGAATGGATCCAACGATAT.

For *TSSK4* expression, m*TSSK4*-encoding cDNA was subcloned in pLV[Exp]-EGFP: T2A: Puro vector. The recombinant lentivirus-encoding targets or invalid vectors were generated using standard procedures and purified by glucose density-gradient centrifugation.

The sequence of si*p65*-1: GAGUUUCAGCAGCUCCUGAAC, and siCtrl: GGAGCGCACCAUCUUCUUC, was synthesized from Tsingke. si*p65*-2 siRNA (6339) was directly purchased from CST.

Expression vector encoding GST-HSP90β was subcloned in pET-GST/TEV vector. S255A and S255D mutant were introduced using Mut Express II fast mutagenesis kit (Vazyme) and verified by DNA sequencing.

### Cell culture and stable cell lines’ establishment

MLE-12 was purchased from ATCC and cultured in Dulbecco’s modified Eagle’s medium supplemented with 10% fetal bovine serum, 2 mM glutamine, 100 U/ml penicillin, and 100 mg/ml streptomycin, at 37 °C with 5% CO_2_. All of the cells in experiments have been tested to be mycoplasma free.

To establish *TSSK4*^−/−^ stable cell line, MLE12 cells were transfected with pLV[sh*TSSK4*]-EGFP: T2A: Puro plasmid and selected with puromycin (1.5 μg/ml).

For TSSK4-persistent expressing MLE12 cells (*TSSK4*^+^), *TSSK4*^−/−^ stable cells were transiently infected with lentivirus-mediated pLV[*TSSK4*]-EGFP-expression vector for 48 h. The results of regulated *TSSK4* expression were tested by immunoblotting.

### Animal experiments

C57BL/6 mice were purchased from SPF Biotechnology Co., LTD (SCXK 2016-0002, China). Mice were maintained under controlled conditions (indoor temperature: 22 ± 1 °C and humidity: 40–60%) and a 12 h dark–light cycle in the specific pathogen-free (SPF) facility. Male 6–8-week-old mice were used in this study. All animal procedures were conducted in accordance with the protocols approved by the research ethics committee of the Second Affiliated Hospital of Guangzhou Medical University.

For bleomycin-induced pulmonary fibrotic mice model, C57BL/6 male mice were treated with 3 mg/kg bleomycin or the same amount of sterile saline via intratracheal administration.

For *TSSK4* knockdown in vivo, C57BL/6 male mice were intratracheally infected with lentivirus-mediated sh*TSSK4* or control vectors at the dose of 1.05 × 10^10^ infectious units (IFUs) in a volume of 30 μl per animal, followed with bleomycin-induced pulmonary fibrosis.

For AKT-agonist administration in vivo, bleomycin-induced pulmonary fibrotic mice were daily intraperitoneally injected with SC-79 (40 mg/kg body weight in 10% DMSO) or the same amount of DMSO in the first 4 days.

Body weight was detected every two days. The mice were preeuthanatized on days 7, 14, and 21 after bleomycin induction. Lung tissues were collected and stocked at −80 °C for molecular analysis, including immunoblotting and hydroxyproline assay, according to the manufacturer’s protocol. For pathological analysis, lung lobes were fixed in 4% paraformaldehyde for 12 h. The tissues were sliced to 5 μm thickness for hematoxylin and eosin (HE) staining, Masson’s trichrome staining, and immunohistochemical or immunofluorescence staining, according to the manufacturer’s protocols.

### Clinical IPF-tissue specimens

Lung-tissue slices from IPF patients were obtained from the Biological Sample Bank of State Key Laboratory of Respiratory Diseases and pathological department of the Second Affiliated Hospital of Guangzhou Medical University. The project was approved by the ethics committee of the Second Affiliated Hospital of Guangzhou Medical University.

### Subcellular fractionation and caspase-3 activity assay

To detect subcellular localization of BAD proteins, cells or tissue lysis were performed for cytosol/mitochondrial fractionation with kit (Abcam). Fractions from the same groups were analyzed by immunoblotting. The sum of BAD proteins in different fractions was set as 100%, and the ratio of BAD in different subcellular fractions was analyzed by ImageJ program.

For caspase-3 activity assay, total lysis from cells or tissues was performed with kit using Ac-DEVD-pNA as substrate (Calbiochem).

### Apoptosis assay, mitochondrial membrane potential, and quantitative real-time PCR

For apoptosis analysis, cells were harvested by Trypsin digestion after different treatments as indicated in figure legends. Annexin V/propidium iodide (PI) staining was performed with Annexin V-AF647/PI apoptosis kit (ES science), following with flow cytometric analysis (BD Biosciences).

For mitochondrial membrane potential, the JC-1 Assay Kits were used to stain cells according to the manufacturer’s instruction. The mitochondrial membrane potential was indicated by a fluorescence-emission shift from monomer to polymer and the ratio of mean fluorescence intensity (MFI) was analyzed by flow cytometer.

For quantitative real-time PCR analysis, total RNA was extracted by Trizol reagent from cells or tissues. Total RNA (1 μg) was used for cDNA synthesis, following quantitative PCR analysis with SYBR Green real-time PCR master Mix (Invitrogen). PCR primers were synthesized from Tsingke. GAPDH was used as internal control. The sequences of primers were listed as below:

*TSSK4*: sense, 5′-CGCCAATCTACCAAGCGTG-3′; antisense, 5′-TGCCGTTTAGCAGAGCTGG-3′

*TGF-β1*: sense, 5′-CTCGGGGGCTGCGGCTACTG-3′; antisense, 5′- GGCGTATCAGTGGGGGTCA -3′

*TGF-β2*: sense, 5′-CGAGCGGAGCGAGCAGGAG-3′; antisense, 5′-TAGGAGGGCAACAACATTA-3′

*BMP2*: sense, 5′-GGGACCCGCTGTCTTCTAGT-3′; antisense, 5′- TCAACTCAAATTCGCTGAGGAC-3′

*BMP4*: sense, 5′-GACTTCGAGGCGACACTTCTA-3′; antisense, 5′-GCCGGTAAAGATCCCTCATGTAA-3′

m*TNF-α*: sense, 5′-TCTTCTCATTCCTGCTTGTGG-3′; antisense, 5′-GGTCTGGGCCATAGAACTGA-3′

*Timp1:* sense, 5′-GCAAAGAGCTTTCTCAAAGACC-3′; antisense, 5′-AGGGATAGATAAACAGGGAAACACT-3′

*Col3:* sense, 5′-AACCTGGTTTCTTCTCACCCTTC-3′; antisense, 5′-ACTCATAGGACTGACCAAGGTGG-3′

*Mmp12*: sense, 5′-CTGGGCAACTGGACAACTCAACTC-3′; antisense, 5′-AATGCTGCAGCCCCAAGGAAT-3′

*α-SMA:* sense, 5′-CCCCTGAAGAGCATCGGACA-3′; antisense, 5′-TGGCGGGGACATTGAAGGT-3′

### GST protein pulldown, immunoprecipitation (IP), HSP90β ATPase activity assay, and kinase assay

For GST pulldown, GST-tagged HSP90β proteins (WT, S255A, or S255D mutant) were IPTG (0.1 mM) induced and purified from BL21 bacteria containing prokaryotic expression vectors of GST fusion target proteins. Incubated with different cellular lysis as indicted in figure legends, GST proteins together with binding targets were precipitated by GSH-Sepharose beads and detected by immunoblotting. The amount of GST-tagged proteins in the system was detected by Coomassie brilliant blue staining.

IP assay was performed as previously described^25^. In brief, 100 μg of total cellular lysates were mixed with 30 μL of protein-A Sepharose beads (50% slurry) containing 4 μl of anti-TSSK4 antibody, and rotated at 4 °C overnight. After short centrifuge, pellet complexes were collected to detect the binding proteins by immunoblotting.

For HSP90β ATPase activity assay, HSP90β in cells was immunoprecipitated with anti-HSP90β antibody. The ATPase activity was detected with total ATPase Assay Kit (Nanjing Jiancheng Biotech., A070-1) according to the manufacturer’s protocols.

For TSSK4 kinase assay, TSSK4 proteins were immunoprecipitated from *TSSK4*^*+*^ cells without or with TNF-α treatment as indicated in figure legends. Purified GST-HSP90β proteins (WT or S255A mutant) were used as substrates to incubate with precipitated TSSK4 in the presence of 0.2 mM cold ATP (nonradioactive) in 30 °C for 1 h. Substrate phosphorylation was detected by immunoblotting with antiphosphoserine antibody.

### Dual-luciferase assay

To examine the p65 transcript activity, different fragments of *TSSK4* promoters were subcloned in pGL3 vector containing the firefly luciferase reporter gene. The constructed pGL3-promoter plasmids along with pCDNA3.1–p65 expression vector and Renilla luciferase reporter vector were cotransfected in 293T cells. At 48 h after transfection, firefly and Renilla luciferase activities were detected by the Dual-Light Combined Reporter Gene Assay System (Promega). The RLU (relative light unit) was normalized by the Renilla reporter signaling.

### Statistical analysis

Cellular apoptosis assay, qPCR, hydroxyproline assay, and luciferase assay between two groups were presented as mean ± s.d. Statistical analysis was conducted using two-tailed unpaired Student’s *t* test. For immune-staining data, at least five random fields were picked for ImageJ program analysis, and conducted using two-tailed unpaired Student’s *t* test. For body-weight-loss analysis, the results were interpreted using one-way ANOVA test. For animal experiments, mice with same age and similar body weight were blinded and randomly divided into different groups. At least 5 mice per group to ensure adequate power, and all of the animal data were included for analysis. Similar variation of each group/treatments was estimated before statistical analysis. In all experiments, *p* < 0.05 was considered to be significant.

## Supplementary information

Supplementary figures.

## Data Availability

All data associated with this study are presented in the paper or the Supplementary Materials. The mass spectrometry proteomics data have been deposited to the ProteomeXchange Consortium via the PRIDE partner repository with the dataset identifier PXD027900.
