# Supplementary material for: TSSK4 upregulation in alveolar epithelial type-II cells facilitates pulmonary fibrosis through HSP90-AKT signaling restriction and AT-II apoptosis
Source: Cell Death Dis. 2021 Oct 13;12(10):938. doi: 10.1038/s41419-021-04232-3 (PMC8514558; doi:10.1038/s41419-021-04232-3)
Supplement: Supplementary file 1 — Supplementary figures. [file 41419_2021_4232_MOESM1_ESM.pdf]

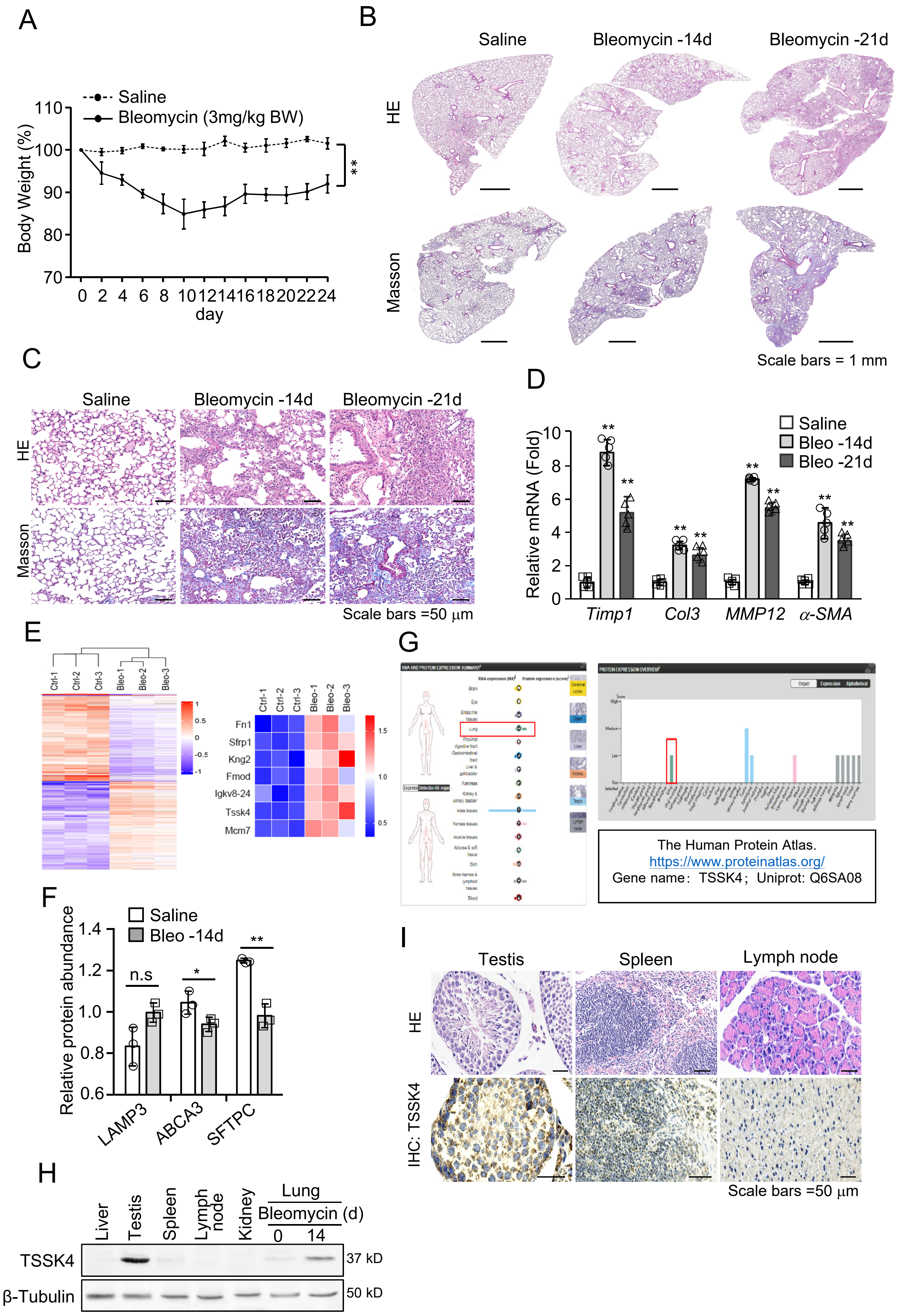
A
Saline
Bleomycin (3mg/kg BW)
Body Weight (%)
day
B
Saline
Bleomycin -14d
Bleomycin -21d
HE
Masson
Scale bars = 1 mm
C
Saline
Bleomycin -14d
Bleomycin -21d
HE
Masson
Scale bars =50 μm
D
Saline
Bleo -14d
Bleo -21d
Relative mRNA (Fold)
Timp1
Col3
MMP12
α-SMA
E
Ctrl-1
Ctrl-2
Ctrl-3
Bleo-1
Bleo-2
Bleo-3
Fn1
Sfrp1
Kng2
Fmod
Igkv8-24
Tssk4
Mcm7
G
The Human Protein Atlas.
https://www.proteinatlas.org/
Gene name: TSSK4; Uniprot: Q6SA08
F
Saline
Bleo -14d
Relative protein abundance
n.s
LAMP3
ABCA3
SFTPC
I
Testis
Spleen
Lymph node
HE
IHC: TSSK4
Scale bars =50 μm
H
Liver
Testis
Spleen
Lymph node
Kidney
Lung
Bleomycin (d)
0
14
TSSK4
β-Tubulin
37 kD
50 kD

Figure S1. TSSK4 is selectively up-regulated in AT II cells of lung fibrosis. C57BL/6 mice were intratracheally treated with bleomycin (3 mg/kg body weight) or same amount of saline for various durations as indicated. (A) Relative body weight loss of model mice were detected for a period of 24 days (n = 10/group). (B and C) H&E staining and Masson's trichrome staining of the representative lungs from fibrosis model mice as indicated. Full view sections, scale bars = 1 mm (B) and expanded fields, scale bars = 50 μm (C). (D) Quantitative mRNA expression of the fibrotic genes including *Timp1*, *Col3*, *MMP12* and *α-SMA* in the lungs were detected through QPCR (n = 5/group). (E and F) Protein levels were measured by quantitative proteomics analysis (n = 3 /group). Cluster analysis and top up-regulated proteins were presented in the Heat-map (E); Relative abundance of AT II associated proteins (LAMP3, ABCA3 and SFTPC) (F). (G) Tissue specific expression of human TSSK4 was analyzed by Atlas database. (H and I) Different tissues (Liver, Testis, Spleen, lymph node and Kidney) from WT mice, and lung tissues from bleomycin (3 mg/kg body weight) induced mice (day 0 and day 14) were performed to analyze the TSSK4 protein level through immunoblotting. α-Tubulin was used as internal control (H); H&E staining and IHC staining with anti-TSSK4 antibody in testis, spleen and lymph node from WT mice as indicated (I). Data in (A, D and F) are presented as means ± s.d. In (A), *** $p<0.001$ by one-way ANOVA test; In (D, F), * $p<0.05$, ** $p<0.01$, n.s $p>0.05$ as analyzed by two-tailed unpaired student's *t* test.

A

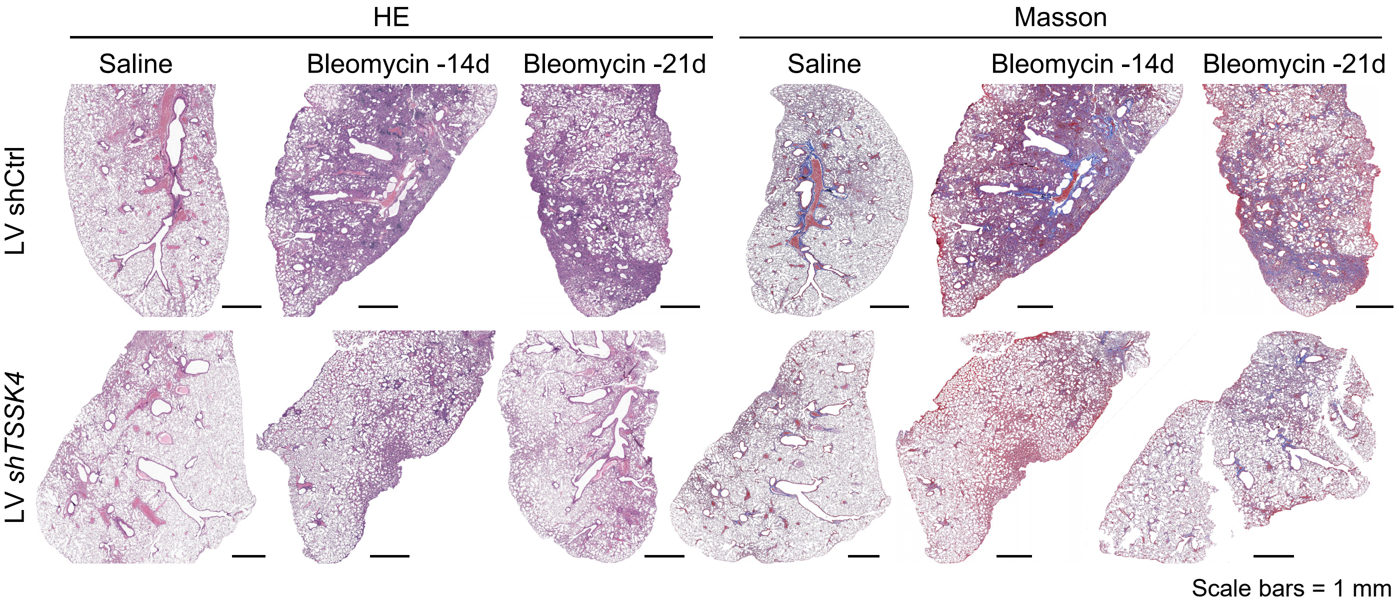

Figure S2. C57BL/6 mice were intratracheally infected with lentivirus mediated sh*TSSK4* or control vectors (1.05×10^{10} infectious units [IFUs] in a volume of 30 μl per animal), and then intratracheally treated with bleomycin (3 mg/kg body weight) or same amount of saline for a period as indicated. Full view sections of H&E staining and Masson's trichrome staining related to (Fig. 2A), scale bars = 1 mm.

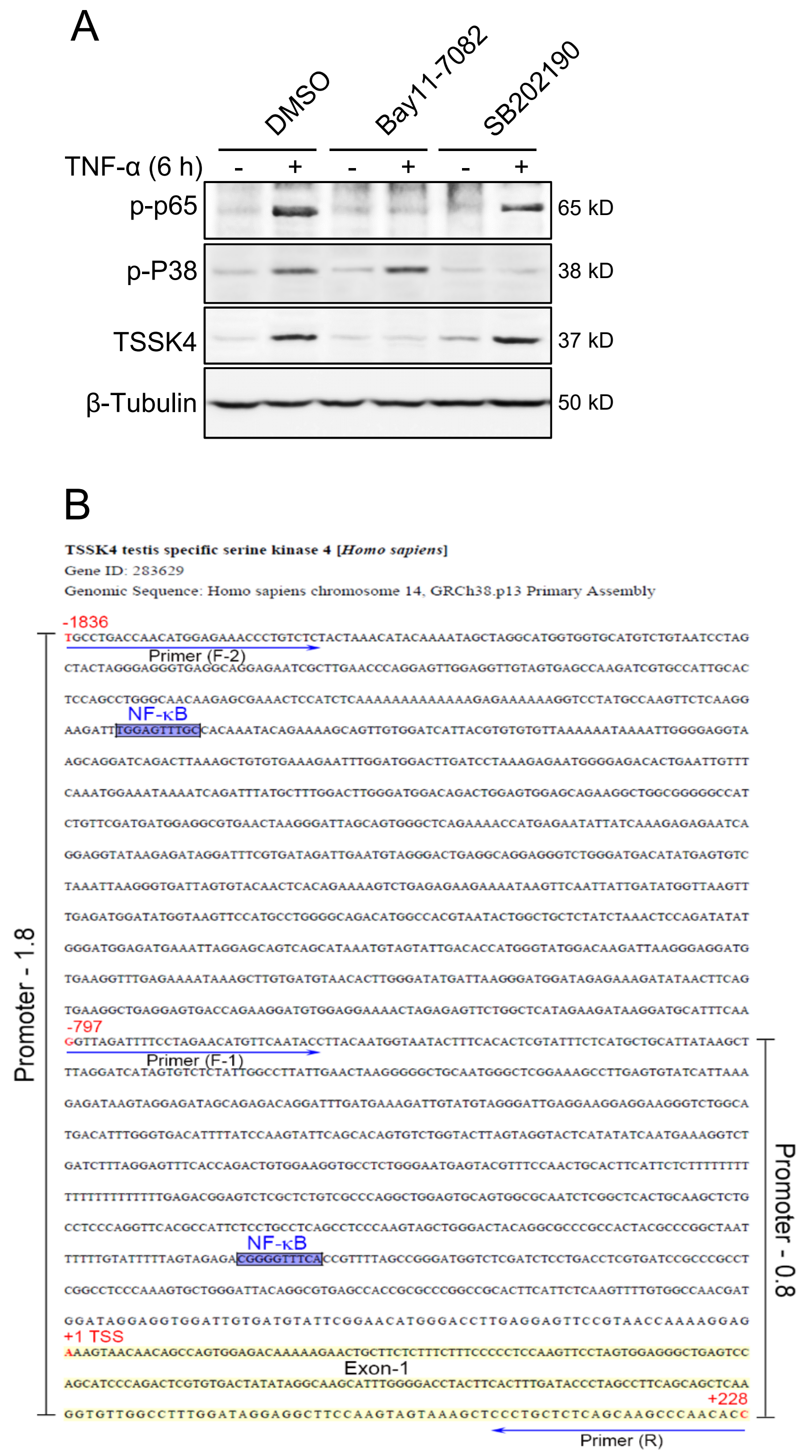

Figure S3. TSSK4 expression is regulated by NF-κB pathway. (A) MLE12 cells were pre-treated with Bay11-7082 (10 μM, for NF-κB inhibition), SB202190 (100 nM, for P38 inhibition) or DMSO for 1 h, followed by stimulation without or with TNF-α (20 ng/ml) for 6 h, as indicated. P65 phosphorylation, P38 phosphorylation, and TSSK4 level were detected by immunoblotting. α-Tubulin was used as internal control. (B) Nucleotide schematic diagram of predicted potential p65 binding sites (marked as blue) in the TSSK4 promoter region. TSS, Transcript Start Site was set as +1 nucleotide. The first exon was marked in yellow. Primers for constructing two promoter fragments (-1.8 and -0.8) were marked out.

A

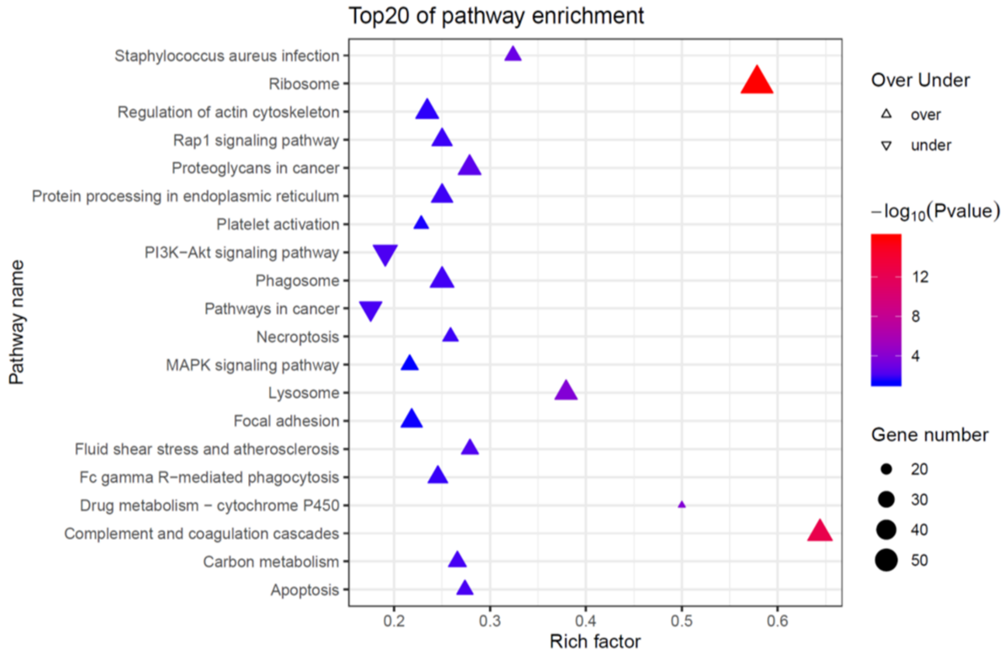

B

Raw data of TUNEL positive ratio of AT II (%)

	View Field	Mouse -1			Mouse -2			Mouse -3			Mouse -4			Mouse -5		
		1	2	3	1	2	3	1	2	3	1	2	3	1	2	3
Bleomycin	0 d	1.2	5.3	2.4	3.1	4.3	5.6	3.1	2.8	1.7	3.3	4.6	5.7	3.9	3.1	6.3
	13 d	37.5	25.3	15.7	16.7	32.8	35.9	23.4	19.2	24.5	39.5	16.5	35.5	35.8	19.4	41.3
	21 d	48.7	34.5	84.3	57.4	39.5	45.2	36.6	41.4	25.4	46.3	39.7	34.1	29.3	35.2	21.8

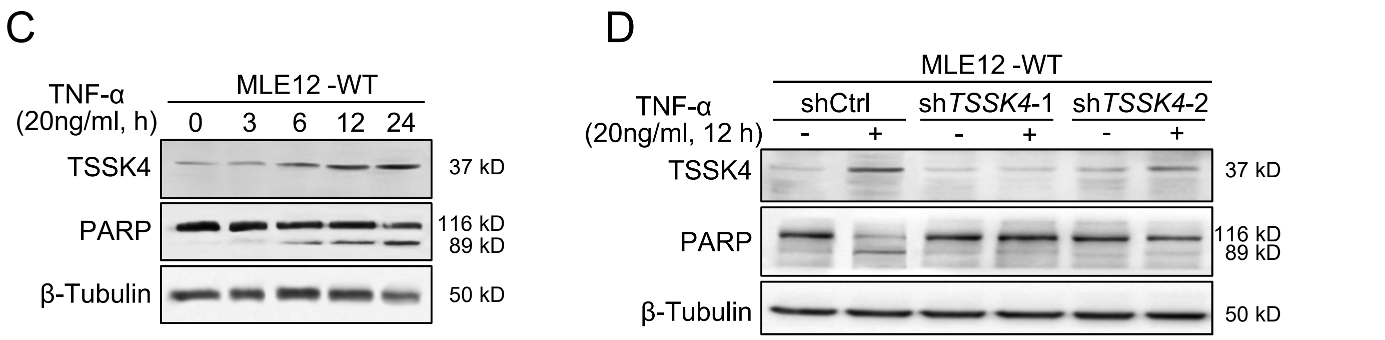

Figure S4. (A) Protein levels were measured by quantitative proteomics analysis as mentioned in (Fig. 1C). KEGG signaling pathway enrichment analysis and top 20 of pathway enrichment were presented in bubble diagram (n = 3 /group). (B, relative to Fig. 4B) In bleomycin induced fibrotic mice models, five individual mice of different groups with three random fields of (Fig. 4A) were performed to analyzed the TUNEL positive ratio in total AT II cells. (C) MLE-12 cells were treatment without or with TNF-α (20 ng/ml) for various durations as indicated. Protein levels of TSSK4 and PARP with cleavage part were analyzed by immunoblotting, with α-Tubulin as internal control. (D) MLE-12 cells were transient transfected with scramble shRNA (shCtrl) or two different target sties of sh*TSSK4*, followed by treatment without or with TNF-α (20 ng/ml) for 12 h. Protein levels of TSSK4 and PARP with cleavage part were analyzed by immunoblotting, with α-Tubulin as internal control. Data in (C and D) represent two to three individual experiments with similar results.

A

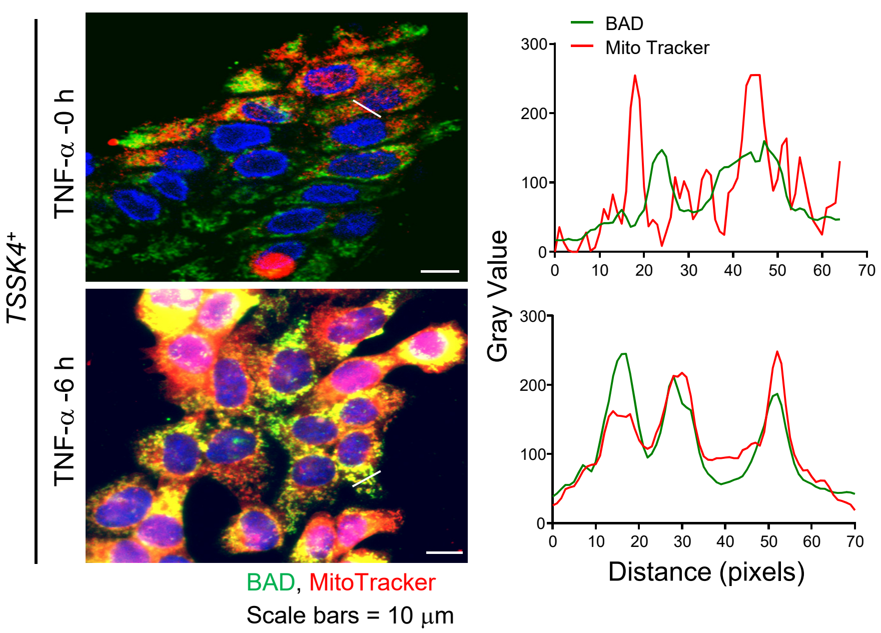

B

Raw data of BAD signaling overlapped with mitochondria

		EXP -1					EXP -2					EXP -3				
View Field		1	2	3	4	5	1	2	3	4	5	1	2	3	4	5
TNF-α	0 h	17.3	11.3	10.8	14.2	7.5	10.4	7.5	6.2	5.5	9.2	8.1	7.8	6.6	8.2	12.5
	6 h	28.4	22.5	20.4	26.5	27.5	26.3	16.6	21.9	23.8	16.8	21.3	20.9	13.8	17.9	22.3

Figure S5. TSSK4 persistent expressing cells (*TSSK4*$^{+}$, as mentioned in Fig. 4E) were stimulated with TNF-α (20 ng/ml) for various durations as indicated. Immunofluorescence staining was performed with anti-BAD antibody, MitoTracker for mitochondria and DAPI for nuclei. (A) Images were taken with confocal microscope (LSM880). The line scan profiles were analyzed by ImageJ program. (B, relative to Fig. 5B) The ration of BAD signaling overlapped with mitochondria were analyzed through ImageJ program in three independent experiments with 5 randomly view fields of each treatment.

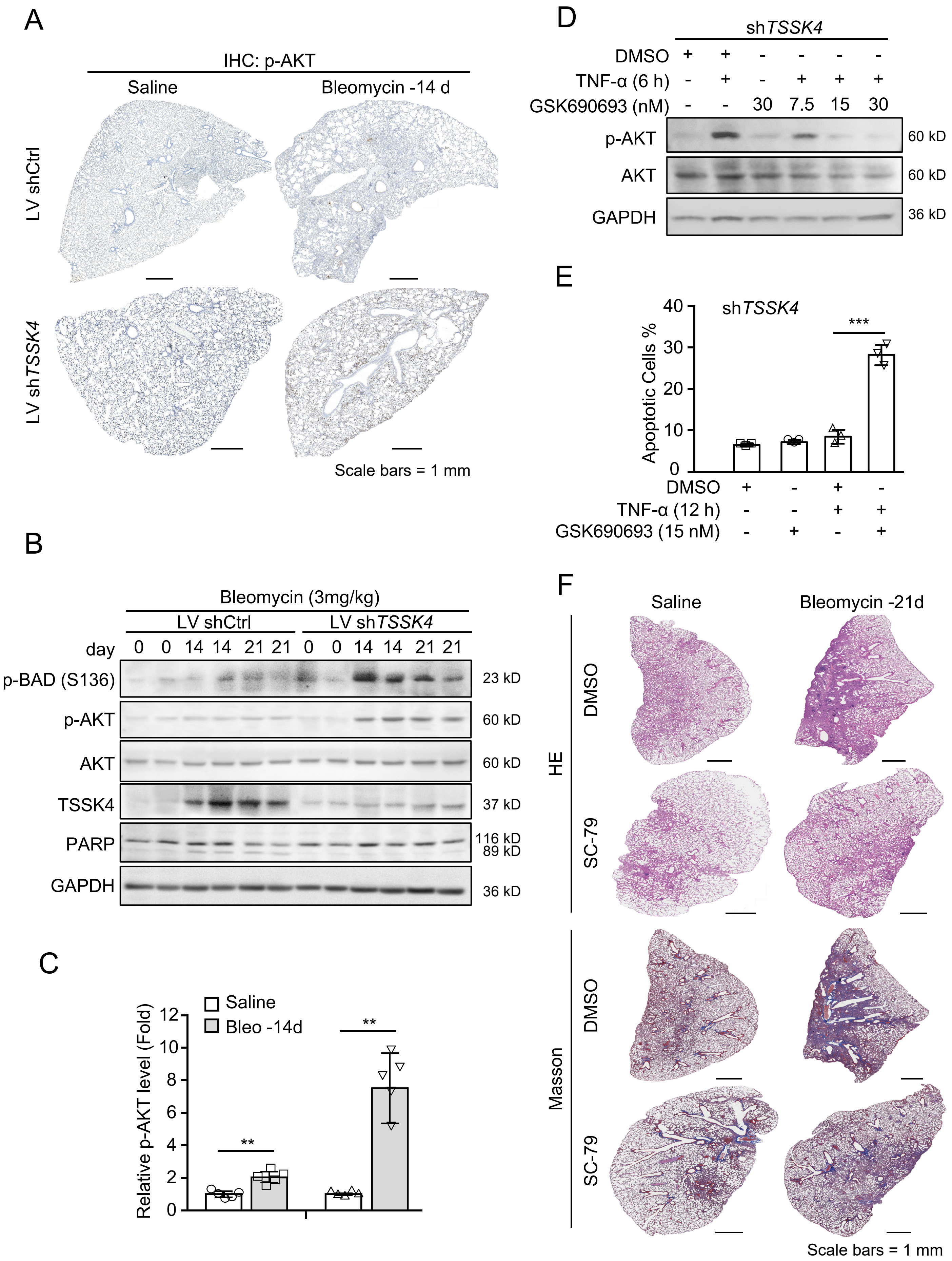
A
IHC: p-AKT
Saline
Bleomycin -14 d
LV shCtrl
LV shTSSK4
Scale bars = 1 mm
B
Bleomycin (3mg/kg)
LV shCtrl
LV shTSSK4
day 0 0 14 14 21 21 0 0 14 14 21 21
p-BAD (S136)
p-AKT
AKT
TSSK4
PARP
GAPDH
23 kD
60 kD
60 kD
37 kD
116 kD
89 kD
36 kD
C
Saline
Bleo -14d
Relative p-AKT level (Fold)
0 2 4 6 8 10 12
**
**
D
shTSSK4
DMSO + + - - - -
TNF-α (6 h) - + - + + +
GSK690693 (nM) - - 30 7.5 15 30
p-AKT
AKT
GAPDH
60 kD
60 kD
36 kD
E
shTSSK4
Apoptotic Cells %
0 10 20 30 40
***
DMSO + - + -
TNF-α (12 h) - - + +
GSK690693 (15 nM) - + - +
F
Saline
Bleomycin -21d
HE
Masson
DMSO
SC-79
DMSO
SC-79
Scale bars = 1 mm

Figure S6. TSSK4 restrains AKT activation to consequently promote AT II apoptosis in vitro and in vivo. (A to B) C57BL/6 mice were intratracheally infected with lentivirus mediated sh*TSSK4* or control vectors (1.05×10^{10} infectious units [IFUs] in a volume of 30 ul per animal), and then intratracheally treated with bleomycin (3 mg/kg body weight) or same amount of saline for a period as indicated. Representative lungs were analyzed by IHC staining with anti-p-AKT antibody (related to Fig. 6A), scale bars = 1 mm (A); Immunoblot analysis of BAD phosphorylation (S136), AKT phosphorylation with its original protein, TSSK4, and PARP with cleavage part in different lung tissue lysis as indicated (B); Densitometric analysis of immunoblotted p-AKT (n=5) (C). (D and E) *TSSK4* knock down (sh*TSSK4*) cells were pre-treated with DMSO or different doses of GSK690693 (for AKT inhibition) for 1 h, followed by stimulation without or with TNF-α (20 ng/ml) for 6 h or 12 h, as indicated. AKT Phosphorylation with its original protein level were detected by immunoblotting (D); Apoptotic cells were detected by Annexin V/Propidium iodide (PI) staining and analyzed by flow cytometry (E). (F) Full view sections of H&E staining and Masson's trichrome staining related to (Fig. 6H), scale bars = 1 mm. Data in (C and E) are presented as means ± s.d. ** $p<0.01$, *** $p<0.001$, as analyzed by two-tailed unpaired student's *t* test. Data in (B and D) represent two to three individual experiments with similar results.
